# Systematic review of melanoma incidence and prognosis in solid organ transplant recipients

**DOI:** 10.1186/2047-1440-3-10

**Published:** 2014-05-06

**Authors:** Erin Dahlke, Christian Alexander Murray, Jessica Kitchen, An-Wen Chan

**Affiliations:** 1 Division of Dermatology, Department of Medicine, Sunnybrook Health Sciences Centre, University of Toronto, 2075 Bayview Avenue, M1-700, Toronto M4N 3 M5, ON, Canada; 2 Women’s College Hospital, Division of Dermatology, Department of Medicine, University of Toronto, 76 Grenville St, Toronto M5S 1B2, ON, Canada; 3 Women’s College Research Institute, 7th floor, University of Toronto, 790 Bay St, Toronto M5G 1N8, ON, Canada

**Keywords:** Melanoma, Cancer, Malignancy, Neoplasia, Transplant, Incidence, Prognosis, Registry, Epidemiology

## Abstract

Cutaneous melanoma carries the potential for substantial morbidity and mortality in the solid organ transplant population. We systematically reviewed the literature published from January 1995 to January 2012 to determine the overall relative risk and prognosis of melanoma in transplant recipients. Our search identified 7,512 citations. Twelve unique non-overlapping studies reported the population-based incidence of melanoma in an inception cohort of solid organ transplant recipients. Compared to the general population, there is a 2.4-fold (95% confidence interval, 2.0 to 2.9) increased incidence of melanoma after transplantation. No population-based outcome data were identified for melanoma arising post-transplant. Data from non-population based cohort studies suggest a worse prognosis for late-stage melanoma developing after transplantation compared with the general population. For patients with a history of pre-transplant melanoma, one population-based study reported a local recurrence rate of 11% (2/19) after transplantation, although staging and survival information was lacking. There is a need for population-based data on the prognosis of melanoma arising pre- and post-transplantation. Increased incidence and potentially worse melanoma outcomes in this high-risk population have implications for clinical care in terms of prevention, screening and reduction of immunosuppression after melanoma development post-transplant, as well as transplantation decisions in patients with a history of pre-transplant melanoma.

## Introduction

Important advances in solid organ transplantation have led to improved patient survival over recent decades. With prolonged survival, the long-term complications of immunosuppressive therapy have become increasingly important. Decreased immune surveillance after transplant leads to a three- to fourfold increased risk of malignancy compared to the general population [1,2].

Skin cancer is the most common form of post-transplant malignancy, particularly squamous cell carcinoma (relative risk 14 to 82) [3-6]. Although the incidence of melanoma is increased to a lesser degree than for squamous cell carcinoma, the potential for melanoma metastasis can introduce significant morbidity and mortality. Previous studies of melanoma in the transplant population have often had low numbers of cases, which precludes precise estimation of relative risk and produces variable estimates across studies. In addition, data on clinical outcomes such as metastasis or mortality are scarce for melanoma arising pre- and post-transplantation.

With its increased incidence and significant metastatic potential, melanoma has important implications for the care of transplant recipients. We aim to systematically review the published literature to determine the overall relative risk and prognosis of melanoma in solid organ transplant recipients.

## Materials and methods

On 12 January 2012, electronic literature searches were conducted in Ovid MEDLINE (1946 to week 1 of January 2012, and In-Process and Other Non-Indexed Citations from 1946 to the present) and EMBASE (1980 to week 2 of 2012) to identify eligible studies using a comprehensive search strategy developed in consultation with an information specialist. The search terms consisted of the following: cancer/neoplas*/tumor/tumour/malignan*/carcin*, or melanoma (expanded), and transplant (organ/kidney/renal/heart/cardiac/liver/hepat/lung/pulmonary/pancreas/intestine/spleen) and cohort/incidence/prevalence/prognosis.

Studies were limited to those published after 1995 in English and French. We included studies that estimated the relative risk or reported clinical outcomes (stage, recurrence, metastasis or death) of melanoma in a population-based inception cohort of solid organ transplant recipients (kidney, liver, heart, lung, pancreas or intestine). Studies were not excluded based on a quality assessment.

We initially screened all titles and abstracts to exclude articles that were clearly ineligible. We then reviewed full-text articles for all remaining citations. Reference lists from relevant articles were also reviewed for relevant articles. When needed, we obtained additional data from the study authors by email correspondence. If there were studies with more than a 50% overlap in their patient populations (based on country, year of transplant, type of graft and data source), we included data from only the most recent or inclusive study.

We pre-specified three main systematic review outcomes: the incidence and relative risk of melanoma diagnosed after solid organ transplantation compared to the general population; the outcomes of melanoma diagnosed after solid organ transplantation (post-transplant melanoma), including recurrence, metastasis and mortality (overall and melanoma-specific); and the outcomes of melanoma diagnosed prior to transplantation (pre-transplant melanoma).

For articles reporting incidence data, the pooled standardized incidence ratio (SIR) and 95% confidence interval (CI) were calculated using weights from a random effects model. The variation in effect sizes attributable to heterogeneity was quantified using the I-squared statistic. To explore potential sources of heterogeneity, we used meta-regression to adjust for the graft organ type (renal/liver versus heart/lung) and the most recent year of transplantation included in the study cohort (>2000 versus ≤2000). All statistical analyses were performed using Stata 12.1.

## Results

**Figure 1:**
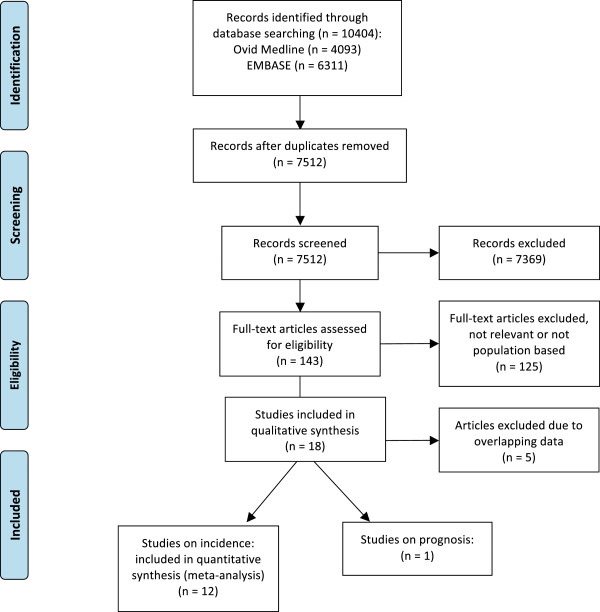
Flow diagram.

The literature search identified 4,093 citations from Ovid MEDLINE and 6,311 from EMBASE. After removing duplicates, 7,512 unique citations were identified. We eliminated 7,369 citations based on title or abstract. A full-text review of the remaining 143 articles led to the further exclusion of 125 articles, most commonly because the studies were not population-based (Figure 1).

We identified 17 studies that reported the incidence of melanoma in a population-based cohort of solid organ transplant recipients [3-19]. No eligible studies of post-transplant melanoma outcomes were found. One population-based study reported the outcomes of pre-transplant melanoma. We requested and obtained additional information from the primary author for two studies through email correspondence [12,20]. (Y Jiang, MD, 7 March 2011, and J Brewer, MD, 24 February 2012).

### Incidence of post-transplant melanoma

Among the 17 population-based studies reporting melanoma incidence after transplant, five were excluded to avoid double counting of data that overlapped significantly with other studies [11,14,15,17,18]. Two Swedish studies collected data from the Swedish National In-patient Registry for patients transplanted between 1970 and 1994 [14] and between 1970 and 1997 [6]; we excluded the older cohort. A Norwegian study that reported skin cancer incidence in heart and renal transplant recipients [15] was excluded due to significant overlap with a larger study [10] that reported cancer risk after renal transplantation in all Nordic countries. We excluded two Australian [17,18] studies that overlapped with a more recent study of a larger cohort of renal transplant recipients in Australia and New Zealand [7]. Finally, two studies from the United States used overlapping datasets; we included the more recent and inclusive study [11,19].

Characteristics of the 12 included studies are listed in Table 1. The study populations were for kidney (*N* = 5), liver (*N* = 2), heart (*N* = 1) and various solid organ transplants (*N* = 4) performed in North America, Europe, Australia and New Zealand. There was less than 50% overlap of populations in three included studies from Sweden [6], Denmark [5] and the Nordic countries [10].

**Table 1:** Characteristics of 12 included studies reporting relative incidence of post-transplant melanoma

**First author (publication year)**	**Country**	**Graft type**	**Transplant years**	**Population size**	**Follow-up time**
Jiang [8]	Canada	Heart	1981–1998	1,703	10,369 person-years
Webster [7]	Australia, New Zealand	Renal	1963–2004	15,183	130,186 person-years; median 7.2 years
Birkeland [10]	Nordic^a^	Renal	1964–1982	5,692	32,392 person-years
Villeneuve [16]	Canada	Renal	1981–1998	11,033	81,237 person-years
Moloney [3]	Ireland	Renal	1986–2001	1,558	Median 5.7 years
Bastiaannet [9]	Netherlands	Renal	1989–2003	1,125	8165 person-years; mean 7.3 years
Aberg [13]	Finland	Liver	1982–2005	540	3222 person-years; mean 6.3 years
Jiang [12]	Canada	Liver	1983–1998	2,034	10,371 person-years
Adami [6]	Sweden	Renal, liver, heart, lung	1970–1997	5,931	40,360 person-years; mean 6.8 years
Jensen [5]	Denmark	Renal, liver, heart, lung	1977–2006	5,279	35,615 person-years; median 5 years
Collett [4]	United Kingdom	Renal, liver, heart, lung	1980–2007	37,617	Median 16 years
Engels [19]	United States	Renal, liver, heart, lung	1987–2008	175,732	775,147 person-years

^a^Denmark, Finland, Norway and Sweden.

Regional cancer registries were the main data source for identifying melanoma diagnoses. All studies reported rates of melanoma in transplant recipients compared to their respective age-, sex- and time-matched general population rates. One study also accounted for race [19].

**Figure 2:**
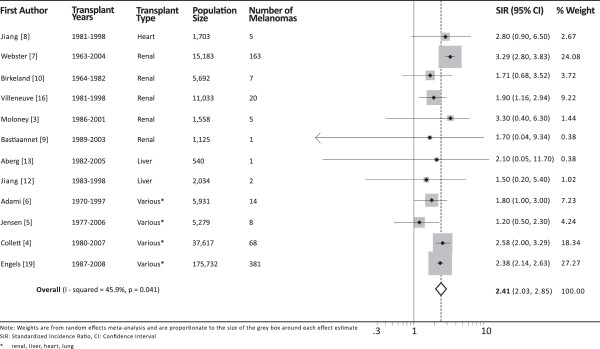
**Forest plot of standardized incidence ratios for 12 included studies.** CI, confidence interval; SIR, standardized incidence ratio.

Overall, transplant recipients have a pooled estimate of 2.4 times (95% confidence interval, 2.0 to 2.9) the risk of melanoma compared to the general population (Figure 2). The overall I-squared was 46% (*P* = 0.04), indicating moderate heterogeneity between studies. Adjusting for the type of organ graft and the most recent year of transplant in the cohort reduced the I-squared value to 0%. Studies of renal or liver transplant recipients had an absolute increase in SIR of 0.29 compared to studies that included heart or lung transplant recipients (*P* = 0.01). Studies that included patients transplanted after the year 2000 had an increase in SIR of 0.41 compared to older studies (*P* = 0.03).

### Prognosis of post-transplant melanoma

Our search identified no population-based studies reporting data on outcomes of *de novo* melanoma arising post-transplantation. We identified ten retrospective, non-population-based cohort studies that compiled data from various sources [20-29]. As many of these papers present overlapping data, the most summative articles are described in Table 2.

**Table 2:** **Non-population-based retrospective cohort studies reporting prognosis of patients with *de novo *** **post-transplant melanoma**

**First author (publication year)**	**Location**	**Transplant data source**	**Number of cases of melanoma**	**Staging data (number of patients with data)**	**Outcomes**
Brewer [20]^a^	United States	Mayo Clinic databases, OPTN, IPITTR	638	AJCC pathologic stage (67 tumors)	Worse 3-year, cause-specific survival for Breslow 1.51 to 3.0 mm and Clark level III or IV in transplant recipients versus controls.
				Breslow depth (123)	
Frankenthaler [21]	United States	BIDMC Cutaneous Oncology Program database	4 (renal transplant)	AJCC stage (19)	Similar relapse rates but worse overall survival in immunosuppressed group versus controls.
			15 (autoimmune disease)		
					Similar stage distribution at diagnosis compared to general melanoma database.
Matin [26]	Europe	SCOPE, AJCC	89	Breslow depth (83)	Worse overall survival for Breslow >2 mm in transplant patients versus controls (HR 11.5, 95% CI 3.6 to 36.8), but not found for Breslow ≤2 mm (HR 1.5, 95% CI 0.31 to 6.9).
				Clark level (82)	
				Ulceration (79)	
					14 patients with metastasis.
Le Mire [22]	United Kingdom	Oxford renal transplant unit	10	Breslow depth (10)	1 patient died from metastatic melanoma (Breslow 4.5 mm).
				Clark level (10)	
				Ulceration (10)	No recurrence in other cases (all <1 mm).
Veness [23]	Australia	St Vincent Hospital, Sydney	8	Regional lymph node status (2)	4 deaths from metastasis.
Lévêque [24]	France	9 centers	17	Breslow depth (16)	4 deaths from metastasis
				Clark level (16)	
				Ulceration (16)	

^a^Overlap with three prior studies [25,27,28].

AJCC, American Joint Committee on Cancer; BIDMC, Beth Israel Deaconess Medical Center; CI, confidence interval; HR, hazard ratio; IPITTR, Israel Penn International Transplant Tumor Registry (previously Cincinnati transplant tumor registry); OPTN, Organ Procurement and Transplantation Network; SCOPE, Skin Care in Organ Transplant Patients, Europe.

In the largest study reporting on post-transplant melanoma outcomes, Brewer *et al*. retrospectively identified 638 cases of post-transplant melanoma in the United States collected from three Mayo Clinic databases (Florida, Rochester and Arizona), the Organ Procurement Transplant Network (OPTN) and the Israel Penn International Tumor Transplant Registry (IPITTR). They found that overall survival rates were worse in the transplant population compared to the general population based on Surveillance, Epidemiology and End Results (SEER) data. In particular, patients with melanomas with a Breslow depth of 1.51 to 3.00 mm and Clark levels III/IV had significantly worse outcomes compared with the expected survival rates in the general population [20]. A number of earlier publications also reported from these same databases [25,27-29].

In the second largest non-overlapping study, Matin *et al.* reported worse outcomes for late stage (T3/T4) melanoma in transplant recipients compared to the general population [26]. This group analyzed cases from the Skin Care in Organ Transplant Patients, Europe network (SCOPE) database, which collects data from 14 transplant dermatology clinics. They compared 81 cases of melanoma in transplant patients to controls matched by age, sex, tumor thickness and ulceration, and found worse outcomes in transplant patients with stage T3/T4 tumors (>2 mm thick). They found outcomes similar to the controls for early stage (T1/T2) melanomas. The hazard ratio for T3/T4 stage tumors was 11.49 (3.6 to 36.8).

Frankenthaler *et al*. reported on outcomes of 19 melanoma patients who were taking immunosuppressive therapies for renal transplantation (4 patients) or autoimmune conditions (15 patients). Each case was matched to three non-immunosuppressed controls with a similar age, sex, stage and location of primary tumor. They found no significant difference in relapse rates but worse overall survival in the immunosuppressed group, suggesting a more aggressive clinical course [21]. Other smaller, uncontrolled cohort studies have shown variable findings (Table 2).

### Post-transplantation prognosis of pre-transplant melanoma

Our search identified one population-based, retrospective, uncontrolled cohort study reporting on post-transplant outcomes of melanoma diagnosed prior to solid organ transplantation (Table 3) [30]. Chapman *et al.* reported data on cancers (excluding non-melanoma skin cancers) diagnosed before renal transplantation that subsequently recurred post-transplant. From the Australian and New Zealand Dialysis and Transplant Registry (ANZDATA), 19 of 11,894 patients who received renal transplants from 1963 to 1999 had a history of pre-transplant melanoma. Two of the nineteen patients (11%) had melanoma recurrence after transplant. No data on staging or post-transplant metastasis were available.

**Table 3:** Uncontrolled cohort studies reporting post-transplant outcomes of melanoma diagnosed prior to solid organ transplantation

**First author (publication year)**	**Population based**	**Location**	**Transplant data source**	**Number of patients with pre-transplant melanoma**	**Staging data (number of patients with data)**	**Post-transplant outcomes (number of patients)**
Chapman [30]	Yes	Australia, New Zealand	ANZDATA	19	Not available	Recurrence (2)
Brewer [20]^a^	No	United States	Mayo Clinic databases, OPTN, IPITTR (1967–2007), SEER	59	Breslow depth (15)	Median post-melanoma follow-up of 10.5 years:
						Local recurrence (0)
						Nodal metastasis (1)
						Lung metastasis (1)
Matin [26]	No	Europe	SCOPE network (14 clinics)	9	Breslow depth (6)	No deaths after median post-melanoma follow-up of 14 years

^a^Overlap with two prior studies [25,28].

ANZDATA, Australian and New Zealand Dialysis and Transplant Registry; IPITTR, Israel Penn International Transplant Tumor Registry; OPTN, Organ Procurement and Transplantation Network; SCOPE, Skin Care in Organ transplant Patients, Europe; SEER, Surveillance, Epidemiology and End Results.

We also identified two non-population based, retrospective, uncontrolled cohort studies (Table 3) [20,26]. Brewer *et al*. examined the post-transplant outcomes of 59 cases of pre-transplant melanoma collected from the IPITTR, OPTN and Mayo Clinic databases [20]. Breslow depth was available for a subset of 17 cases. They reported no recurrences and two melanoma metastases with a mean follow up of 10.5 years. This study encompassed data from two previous reports [25,28], including data from IPITTR that originally suggested that 6 of 31 patients with pre-transplant melanoma died from post-transplant recurrences [28]. However, these findings could not be replicated by Brewer *et al*., whose study excluded melanoma diagnoses from IPITTR that were not histopathologically confirmed (personal communication, J Brewer, MD, 24 February 2012).

Matin *et al*. reported data voluntarily provided by physicians in the SCOPE network database (Table 3). Breslow depth was available for six of nine patients with pre-transplant melanoma. No post-transplant deaths were noted after 3.3 to 42 years of post-melanoma follow-up (median 14 years) and 0.5 to 10.2 years of post-transplant follow-up (median 5 years) [26].

## Discussion

This is the first systematic review of melanoma incidence and outcomes in the solid organ transplant population. Compared to the general population, there is a 2.4-fold increased incidence of melanoma in the transplant population. No population-based outcome data were identified for melanoma arising post-transplant. Data from non-population-based cohort studies suggest a worse prognosis for late-stage melanoma developing in a transplant population versus a general population. For melanoma arising prior to transplantation, one uncontrolled population-based study found an 11% local recurrence rate post-transplant, although staging and survival data were lacking.

Immune function plays a prominent role in the biological response to melanoma [31]. The increased incidence of melanoma in the transplant population is likely due to decreased immune surveillance, although the oncogenic potential of systemic immunosuppressant medications may also play a role. Reduced immune surveillance may also lead to poorer outcomes in melanoma arising pre- and post-transplant, but clinical data are limited. Late transmission of donor melanoma after up to 32 years of melanoma-free survival in the host suggests that immunosuppression can promote activation of previously dormant melanoma cells [32]. Melanoma regression with removal of immunosuppression has also been described [33], and many of the systemic treatments for metastatic melanoma are immune-activating therapies [34].

In transplant candidates with a history of melanoma, the risk of post-transplant recurrence and metastasis has important implications for the decision to pursue transplantation. The quality of data available to guide this decision is currently limited to uncontrolled, non-population-based reports that lack staging information. Otley *et al*. proposed a consensus-based framework of waiting time prior to consideration of transplantation, based on melanoma stage [35]. The risks and benefits of pursuing transplantation versus continued dialysis or organ failure in patients with a history of melanoma need to be weighed on a case-by-case basis.

Our study has several limitations. Firstly, our review is limited by the quality of the included studies. Only one incidence study adjusted the melanoma rates for race, an important risk factor for skin cancer [19]. This could affect the estimates of relative risk if the racial composition differed between transplant and general populations. Data on the level of immunosuppression were also not available for the identified studies, with inevitable heterogeneity of medications and dosing within each transplant population and between studies. Furthermore, staging information was uniformly lacking, which precluded evaluation of the severity of melanoma at the time of diagnosis in the transplant population versus the general population, and the impact of stage on melanoma outcomes. Finally, the overall heterogeneity of the included incidence studies was moderate, as reflected by the I-squared value of 46%. We explored two potential sources of heterogeneity and found that the graft type and cohort year accounted for all of the between-study variability.

## Conclusion

Our systematic review found that there is a 2.4-fold increased incidence of melanoma in the transplant population compared to the general population. Non-population-based data suggest a worse prognosis for late-stage melanoma developing in the transplant population versus the general population. These findings have several implications for clinical practice and research. The significantly increased overall risk of melanoma arising post-transplant means that these patients warrant a multi-pronged approach to primary and secondary prevention, including regular full skin examinations for cancer screening, a low biopsy threshold for suspicious pigmented lesions, and continual education on the importance of sun avoidance and protection. Our study has also identified an important knowledge gap that should be addressed in future research. Population-based studies that account for melanoma stage and risk factors are needed for patients and clinicians to understand better the prognosis of pre- and post-transplant melanoma. These data would help to inform treatment decisions, as tumors known to have a poorer prognosis may require more aggressive management, including significant reduction or discontinuation of immunosuppression. Robust outcome and staging data would also help to inform the decision to pursue, avoid or delay transplantation in patients with a history of melanoma.

## Abbreviations

AJCC: American Joint Committee on Cancer; ANZDATA: Australian and New Zealand Dialysis and Transplant Registry; BIDMC: Beth Israel Deaconess Medical Center; CI: confidence interval; IPITTR: Israel Penn International Tumor Transplant Registry; OPTN: Organ Procurement Transplant Network; SCOPE: Skin Care in Organ Transplant Patients, Europe network; SEER: Surveillance, Epidemiology and End Results; SIR: standardized incidence ratio.

## Competing interests

The authors of this manuscript have no conflicts of interest to disclose.

## Authors’ contributions

All authors participated in the design of the study. ED carried out the search. AWC completed the statistical analysis. All authors were involved with analysis and presentation of collected information. ED and AWC drafted the manuscript. ED composed Figure 1. AWC and ED composed Figure 2. ED drafted the tables. CAM and JK revised the manuscript, figures and tables. All authors read and approved the final manuscript.
